# One misdated sequence of rabbit hemorrhagic disease virus prevents accurate estimation of its nucleotide substitution rate

**DOI:** 10.1186/1471-2148-12-74

**Published:** 2012-05-30

**Authors:** Allison L Hicks, Siobain Duffy

**Affiliations:** 1 Department of Ecology, Evolution, and Natural Resources, School of Environmental and Biological Sciences Rutgers, The State University of New Jersey, 14 College Farm Rd, New Brunswick, NJ, 08901, USA

**Keywords:** RHDV, Substitution rate, Tip-calibrated, BEAST, Misdated taxon

## Abstract

**Background:**

The literature is ripe with phylogenetic estimates of nucleotide substitution rates, especially of measurably evolving species such as RNA viruses. However, it is not known how robust these rate estimates are to inaccuracies in the data, particularly in sampling dates that are used for molecular clock calibration. Here we report on the rate of evolution of the emerging pathogen Rabbit hemorrhagic disease virus (RHDV), which has significantly different rates of evolution for the same outer capsid (VP60) gene published in the literature. In an attempt to reconcile the conflicting data and further elucidate details of RHDV ’s evolutionary history, we undertook fresh Bayesian analyses and employed jackknife control methods to produce robust substitution rate and time to most recent common ancestor (TMRCA) estimates for RHDV based on the VP60 and RNA-dependent RNA polymerase genes.

**Results:**

Through these control methods, we were able to identify a single misdated taxon, a passaged lab strain used for vaccine production, which was responsible for depressing the RHDV capsid gene’s rate of evolution by 65%. Without this isolate, the polymerase and the capsid protein genes had nearly identical rates of evolution: 1.90x10^-3^ nucleotide substitutions/site/year, ns/s/y, (95% highest probability density (HPD) 1.25x10^-3^-2.55x10^-3^) and 1.91x10^-3^ ns/s/y (95% HPD 1.50x10^-3^-2.34x10^-3^), respectively.

**Conclusions:**

After excluding the misdated taxon, both genes support a significantly higher substitution rate as well as a relatively recent emergence of RHDV, and obviate the need for previously hypothesized decades of unobserved diversification of the virus. The control methods show that using even one misdated taxon in a large dataset can significantly skew estimates of evolutionary parameters and suggest that it is better practice to use smaller datasets composed of taxa with unequivocal isolation dates. These jackknife controls would be useful for future tip-calibrated rate analyses that include taxa with ambiguous dates of isolation.

## Background

The exponentially accumulating sequence data in GenBank have allowed for the publication of hundreds of nucleotide substitution rate estimates for the rapidly evolving RNA viruses. Within a given viral species, published tip-calibrated Bayesian substitution rate estimates are often highly consistent (e.g. Influenza A virus [1-3] and Rabies virus [4-8]). However, for some viruses, such as rabbit hemorrhagic disease virus (RHDV), there is a significant discrepancy among published substitution rates [9-13].

RHDV is a positive-sense, single-stranded RNA virus of the family *Caliciviridae* (genus *Lagovirus*) and the causal agent of the highly lethal rabbit hemorrhagic disease (RHD). Since the emergence of RHD in China in 1984 [12,14-16], RHDV has spread worldwide and continues to be a growing concern for rabbit meat and fur industries [17,18], as well as a threat to European ecosystems [19]. Heightened surveillance for RHDV has resulted in the identification of rabbit calicivirus (RCV), a nonpathogenic relative of RHDV, in Australia, the United States, and Europe [20-24].

RHDV has mean published substitution rate estimates for the outer capsid (VP60) gene ranging from 5.48x10^-4^ nucleotide substitutions per site per year (ns/s/y) [9] to 2.65x10^-3^ ns/s/y [13], with non-overlapping 95% highest posterior density (HPD) intervals for these lowest and highest estimates. Not surprisingly, there is also significant variation among the time to most recent common ancestor (TMRCA) estimates for RHDV, with estimated mean coalescent ranging from 1917 [12] to 1967 [13]. As RHD was first described in 1984 [12,14-16,25], the oldest TMRCA implies that the coalescent of virulent RHDV antedates the emergence of RHD by almost seven decades. In the absence of an intermediate reservoir host, it is uncommon for the emergence of an acute, virulent virus to be so extensively decoupled from the appearance of its associated disease [3,26-31]. As a result, there has been significant debate over the timing, location, and mechanisms of RHDV ’s emergence [12,14,15,25,32,33].

It is possible that the discrepancy among evolutionary rate estimates for RHDV is partially attributable to variation among datasets (uneven temporal or geographic representation, number of taxa, portion of genome analyzed) and/or subtle methodological variations [34-36]. As this range of evolutionary rates is atypical for a gene of a single viral species, a systematic investigation was undertaken to explain variation among published nucleotide substitution rate and TMRCA estimates for RHDV. A combination of jackknifing controls was used to produce robust rate estimates for the VP60 gene and the first estimated substitution rate for RHDV ’s RNA-dependent RNA polymerase (RdRp) gene. These controls reveal that using one misdated taxon significantly slows the estimated rates, unnecessarily lengthening RHDV ’s TMRCA. We demonstrate the fragility of tip-calibrated evolutionary analyses and propose jackknife control BEAST runs as a way to identify potential misdated taxa.

## Results

### Complete dataset analyses

The best-fitting nucleotide substitution model for the complete VP60 datasets, regardless of whether or not RCV isolates were included, was GTR + I + Γ (general time reversible including corrections for invariant sites and a gamma distribution of rate heterogeneity). For the RdRp, the TrNef + Γ model (equal-frequency Tamura-Nei model including a gamma distribution of rate heterogeneity) was selected for the RHDV dataset, while the SYM + I + Γ model (symmetric six-rate model assuming equal base frequencies, including corrections for invariant sites and a gamma distribution of rate heterogeneity) was selected for the RHDV + RCV dataset. The uncorrelated lognormal clock model was determined to be a significantly better fit than the strict clock model for each of the datasets (log^10^Bayes factors ≫ 2). Additionally, the 95% HPDs of the standard deviation for the uncorrelated lognormal relaxed molecular clock rate estimates excluded zero for all of the demographic models, further rejecting a strict molecular clock for these alignments. The Bayesian skyline demographic model was best-fitting for each of the datasets, though it was not significantly better than the constant or exponentially growing population models. However, there was no significant variation in nucleotide substitution rate or TMRCA estimates among demographic models in any of the full datasets.

There was no significant substitution rate variation between RHDV and RHDV + RCV datasets of either gene (Table 1), and the inclusion of the divergent RCV isolates had no significant effects on age estimates for the virulent RHDV clade or its individual lineages (Table 1, Additional files 1, 2). The subsequent analyses were performed only on the RHDV datasets.

**Table 1:** Complete dataset nucleotide substitution rate analyses

	**Gene**	**N**_**taxa**_	**Substitution Rate (x10**^**-3**^)*	RHDV TMRCA*	Corresponding Year*
RHDV	VP60	65	0.68	77	1932
		(+AY269825)	(0.40-0.97)	(49–113)	(1896–1960)
RHDV	VP60	64	1.91	41	1968
		(−AY269825)	(1.50-2.34)	(31–54)	(1955–1978)
RHDV	RdRp	31	1.90	62	1947
			(1.25-2.55)	(37–89)	(1920–1972)
RHDV + RCV	VP60	104	1.01	90	1919
		(+AY269825)	(0.63-1.44)	(55–142)	(1867–1954)
RHDV + RCV	VP60	103	2.24	45	1964
		(−AY269825)	(1.61-2.95)	(33–60)	(1949–1976)
RHDV + RCV	RdRp	33	2.33	68	1941
			(1.19-3.56)	(43–98)	(1911–1966)

*Mean substitution rate, TMRCA, and corresponding year estimates are shown with upper and lower 95% HPD bounds.

**Figure 1:**
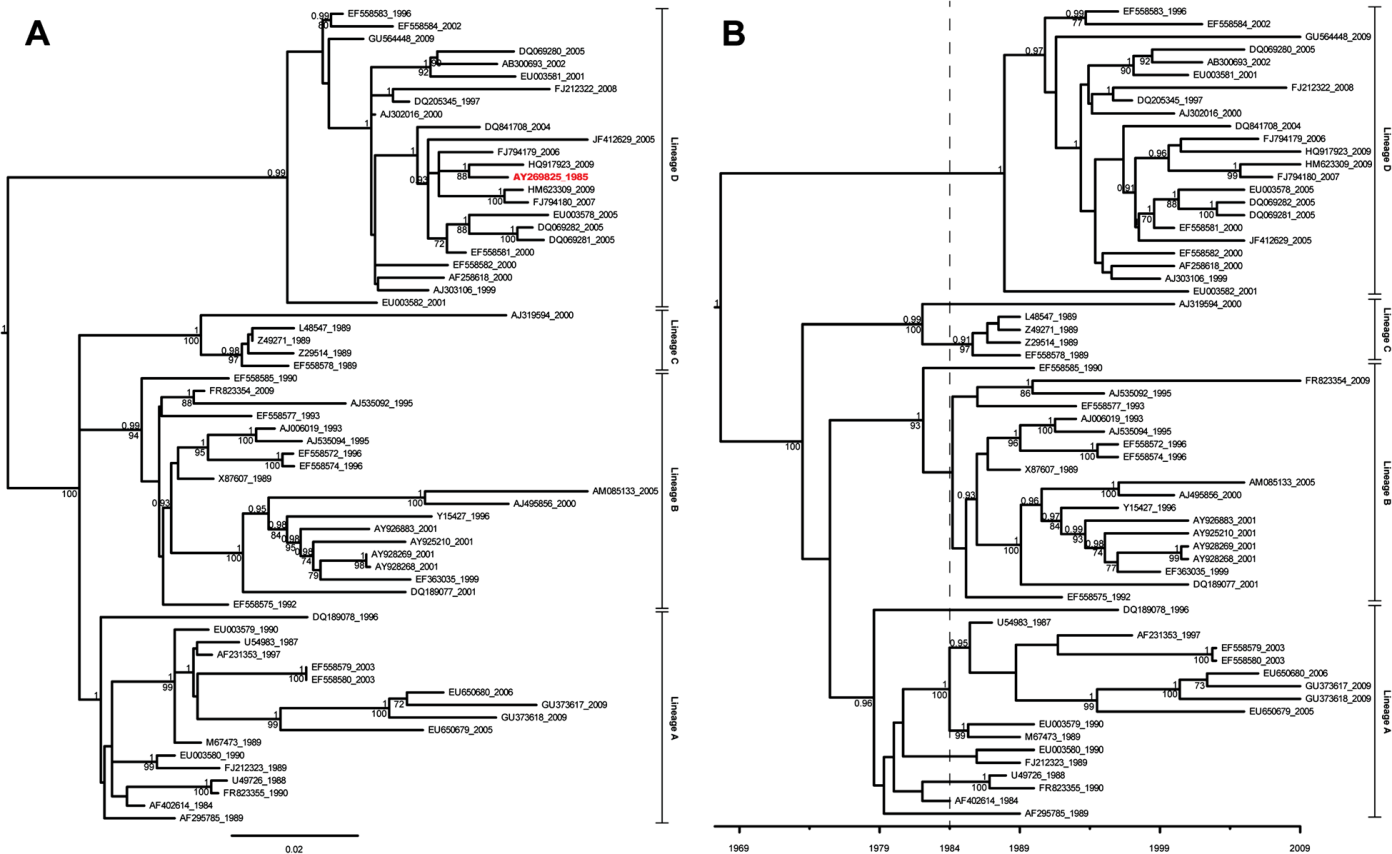
**ML (A) and MCC (B) trees from the complete RHDV VP60 dataset**. The ML tree (**A**) includes AY269825, while the MCC tree (**B**) does not. At the nodes of each tree, posterior probabilities from the BEAST analysis are shown above bootstrap values from the maximum likelihood analysis. Only posterior probabilities greater than or equal to 0.9 are shown, and only bootstrap values greater than or equal to 70 are shown. The dashed line in (**B**) indicates the 1984 emergence of RHD in China.

**Figure 2:**
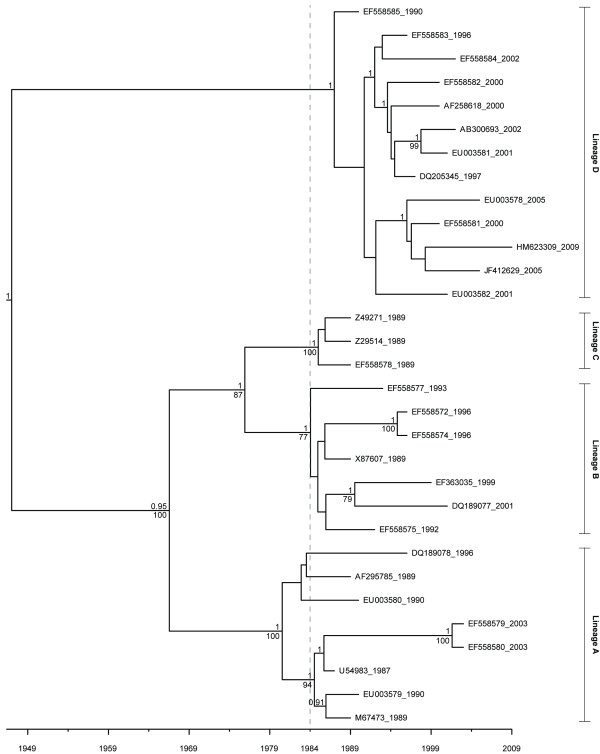
**MCC tree from the complete RHDV RdRp dataset**. At the nodes, posterior probabilities from the BEAST analysis are shown above bootstrap values from the maximum likelihood analysis. Only posterior probabilities greater than or equal to 0.9 are shown, and only bootstrap values greater than or equal to 70 are shown. The dashed line indicates the 1984 emergence of RHD in China.

The maximum clade credibility (MCC) and maximum likelihood (ML) trees for each complete gene dataset were highly congruent (Figures 1, 2). Further, the trees generated for the two different genes were also congruent, with the exception of one taxon, GenBank accession number EF558585, which switched from lineage B in the VP60 tree to lineage D in the RdRp tree (Figures 1, 2). While it has been previously suggested that this taxon has undergone a crossover event at the junction between the RdRp and VP60 genes [37], it was not detected as a potential recombinant in these single gene analyses.

Despite the congruence of tree topology, however, there was a significant difference (non-overlapping 95% HPDs) between the substitution rate estimates from the complete VP60 and RdRp datasets (Table 1), with the VP60 rate estimate paralleling the lower VP60 rates from the literature [9,10,12], and the RdRp rate paralleling the higher published VP60 rate [13].

To assess the extent of temporal structure in the MCC trees, tip-date randomized controls were run. For both genes, the upper 95% HPD interval from the tip-date randomized datasets occasionally overlapped the lower 95% interval of the real dataset substitution rates (Additional file 3). A post-hoc permutation test verified that estimates from the tip-date randomized data sets were different from the substitution rates estimated from the actual dataset (p < 0.06 for VP60, p < 0.02 for RdRp). Root-to-tip regressions showed moderate correlations (VP60: 0.76, RdRp: 0.70). There is statistical support for temporal structure in each gene’s dataset.

### Control analyses

**Figure 3:**
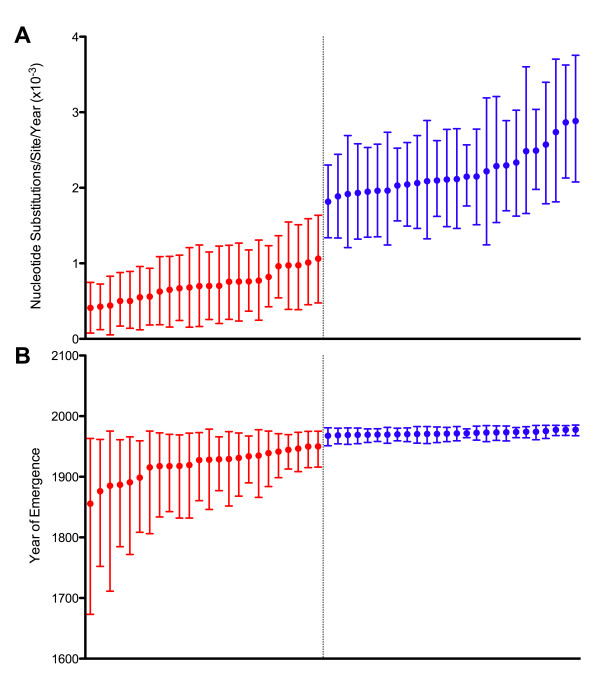
**RHDV nucleotide substitution rates (A) and years of emergence (B) estimated from the VP60 jackknife datasets**. Mean substitution rates are shown with 95% HPD intervals for each of the 50 datasets of 30 random taxa. Estimates produced from datasets that included AY269825 are shown in red, while datasets that did not include AY269825 are shown in blue.

**Figure 4:**
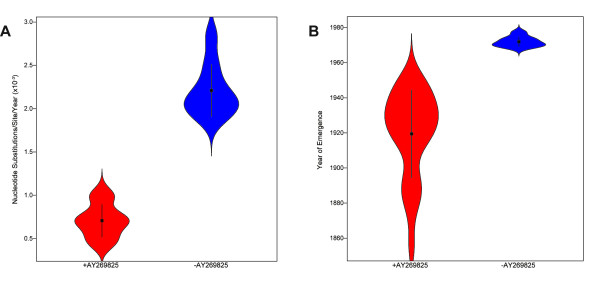
**Violin plots displaying AY269825’s influence on estimated substitution rates (A) and years of emergence (B) for RHDV. Both plots are based on the mean rates and TMRCA from the VP60 jackknife analysis results**. For each plot, datasets represented on the left included AY269825 (“+AY269825”), while datasets represented on the right did not (“-AY269825”). Black rectangles and lines indicate the arithmetic mean and standard deviation of each group of data, respectively, while the shading demonstrates the probability density of each group.

The jackknifed 30 taxa control analysis for the VP60 gene resulted in two distinct groups (Figure 3), roughly corresponding to the two divisions of RHDV rate and TMRCA estimates in literature. By comparing the compositions of the two groups, it was found there was only one taxon (GenBank accession number AY269825) that was present in every dataset from one group and absent from every dataset in the other group. The statistical significance of grouping datasets by the presence or absence of AY269825 is shown in Figure 4. In contrast to the VP60 dataset, the jackknifed 15 taxa control analysis for the RdRp dataset, which did not contain AY269825, yielded one continuous group of substitution rate estimates (Additional file 4).

**Figure 5:**
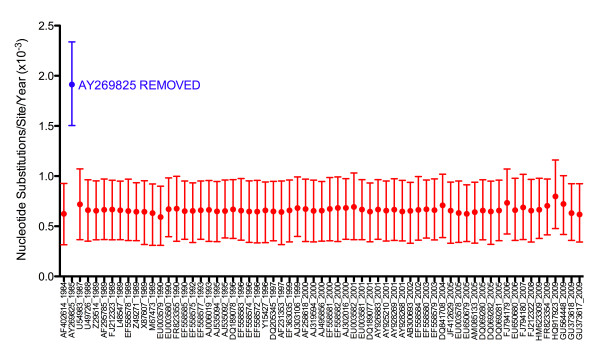
**RHDV nucleotide substitution rates estimated from the VP60 “n-1” jackknife datasets**. Mean substitution rates are shown with 95% HPD intervals for each of the 65 datasets. Each rate estimate is identified on the x-axis by the GenBank accession number and year of isolation of the taxon removed from its dataset. Estimates produced from datasets that included AY269825 are shown in red, while the dataset that excluded AY269825 is shown in blue

The n-1 jackknife control analysis for the VP60 gene further implicated AY269825 as having a significant effect on the estimated substitution rate. Only the removal of AY269825 resulted in a substitution rate estimate that was significantly higher than the other n-1 jackknife datasets (Figure 5) and the complete VP60 dataset (Table 1). Removal of AY269825 also resulted in a TMRCA estimate that was substantially more recent than that from the complete dataset (Table 1). For the RdRp, the substitution rates estimated from the 31 n-1 jackknife datasets were nearly identical to each other and to the complete dataset (Additional file 5).

**Figure 6:**
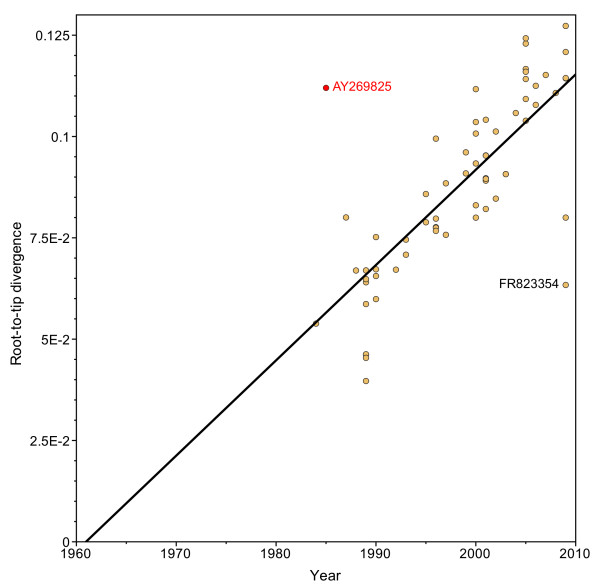
**Regression plot for the complete RHDV VP60 dataset**. Using the best-fitting root, the regression line indicates the relationship between the root-to-tip genetic distance and the isolation date of each taxon. The two outliers (AY269825 and FR823354, with residual values of 0.055 and 0.049, respectively) are indicated by GenBank accession number.

AY269825, more formally “NJ/China/1985,” has been used in previous analyses with 1985 as the year of isolation [10,12,15,23,38,39]. By the leaf-dating method, it was estimated that a more appropriate year would have been 2006 (95% HPD 2003–2009). A root-to-tip regression of the VP60 ML tree, including AY269825, revealed that both AY269825 and FR823354 were potential outliers (highest residuals, Figure 6).

**Figure 7:**
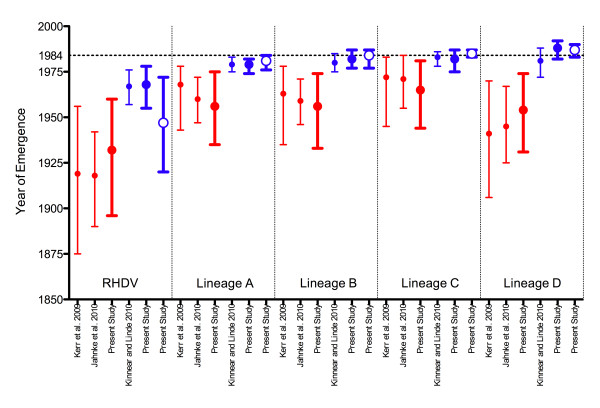
**Estimated years of emergence for RHDV lineages**. Mean years are shown with 95% HPD intervals for the entire virulent RHDV clade and each of its four lineages. Closed circles denote estimates based on the VP60 gene, while open circles denote estimates based on the RdRp gene. Years of emergence estimated in the present study are bolded, while those derived from the literature are not. Estimates based on datasets that included AY269825 are shown in red, while those based on datasets that excluded AY269825 are shown in blue. The source of the MCC tree from which each estimate was inferred is indicated on the x-axis.

Running a fresh BEAST analysis for the complete VP60 dataset without AY269825 revealed that there was no overlap between the HPDs of these substitution rate estimates and those of the dataset with AY269826, regardless of the demographic priors used. By comparing the estimated years of emergence of virulent RHDV and its individual lineages from analyses with and without AY269825 (Table 1, Figure 7), it was revealed that while the inclusion of this taxon inflated TMRCA estimates for all lineages, the most conspicuous effect was on the lineage in which it grouped (lineage D). Removal of FR823354, however, had little effect on the estimated VP60 substitution rate and coalescent (Figure 5; estimated substitution rate 7.04x10^-4^, 95% HPD: 4.14x10^-4^-9.71x10^-4^, MRCA 1932, 95% HPD: 1901–1959).

Removal of AY269825 from the complete VP60 dataset did not change the MCC or ML tree topology (Figure 1). However, the tip-date randomization analysis performed on the VP60 dataset without AY269825 revealed stronger evidence of a temporal signal (no overlap of 95% HPDs from randomized datasets with those of the true dataset; Additional file 3). Similarly, excluding AY269825 from a root-to-tip regression improved the correlation between genetic divergence and time (r = 0.83, compared to 0.76 when AY269825 was included).

## Discussion and conclusions

The notion that inaccurate specification of dates used for molecular rate calibration could produce misleading results is not a novel one [12,35,40-43]. In the context of RHDV in particular, seven partial VP60 sequences were identified as misdated modern contaminants by maximum likelihood analysis [12], but were nonetheless included in one BEAST analysis [9], resulting in the slowest substitution rate published for RHDV. It has also been suspected for some time that certain taxa could have a strong influence over inferred phylogenies, and a number of methods have been developed to identify weak clades in phylogenetic trees [44-47] or the highly influential taxa responsible for weakening phylogenetic relationships [48]. However, there is currently no direct method for identifying the presence of a taxon, such as AY269825, that has significant influence over evolutionary rate and TMRCA estimates, while not altering phylogenetic relationships. Further, the influence of misdated taxa on estimates of evolutionary parameters has yet to be extensively examined or quantified.

The comprehensive jackknifing controls used here demonstrate that the Chinese RHDV isolate NJ/China/1985, GenBank accession number AY269825, was responsible for dragging down the substitution rate estimate for the VP60 gene by 65%. The Chinese-language paper that described this taxon [49] contained important details about the isolation and handling of this strain. The first paragraph of the methods section (see translation below) revealed that, though it was isolated from nature in 1985, it was maintained in the laboratory for vaccine preparation and was likely not sequenced until much later. The 2003 submission date of AY269825 to GenBank is concordant with the lower bound of the age estimated by the leaf-dating method, as well as its grouping with isolates from 2006–2009 in the MCC and ML trees (Figure 1).

"“RHDV NJ85 strain isolate was discovered and characterized byInstitute of Veterinary medicine, Jiangsu Academy of Agricultural Sciences (JAAS), from rabbits raised in an unknown farm in Nanjing City, China, in 1985. Since the discovery, this strain hasbeen maintained in lab rabbits until now. This strain has been usedto prepare potent rabbit hemorrhagic disease vaccine for years Our lab has cloned the gene VP60 in E. coli JM109 and BL21(DE3).”"

The grouping with much more recent isolates could be explained by AY269825’s use in RHDV vaccine production. Whether attenuated or improperly inactivated, the strain could have been released into China, and now this lineage can be isolated from other regions of China and Russia ([50,51]; see GenBank file for HM623309 and FJ794179). This is similar to the lab-escape strain that complicated substitution rate estimation for Influenza A virus [40]. Instead of changing many dates of isolation, however, only AY269825 would have to be assigned a different date (removing the four taxa that grouped with AY269825 did not change the estimated substitution rate or TMRCA of RHDV, data not shown). Previous studies have shown that the long-term rate of viral evolution in the lab can mimic the rate in nature [52], so unlike the 20 years of frozen stasis that the Influenza isolate experienced, AY269825 was changing at a rate similar to its wild relatives.

However, consistent results were obtained by excluding this isolate from analyses altogether. Without AY269825, the TMRCA for the entire virulent RHDV complex and each of its lineages is substantially lower, resolving the much of the debate over its puzzling evolutionary history. Indeed, without this misdated taxon, the ancestor of the entire complex is estimated to have existed between 1955-1978 (Table 1), as few as six years before the 1984 appearance of RHD. These results cannot address whether virulence was a shared trait of the most recent common ancestor of RHDV, or if it evolved independently in multiple lineages. Whenever virulence did evolve, it did not have to go undocumented for several decades [9,12,14,15,23,25,32,33].

While the root-to-tip regression analysis identified AY269825 as potentially deviating from the molecular clock, FR823354, a taxon that did not affect VP60’s evolutionary dynamics (Figure 5, [13]), also had a similarly high residual value (0.055 cf 0.049). This underscores the problem of using congruence with a strict molecular clock as the sole means of assessing the validity of dates of isolation. Deviation from the root-to-tip regression line is expected for viruses with variable rates of evolution, which would be accurately modeled with a relaxed molecular clock BEAST analysis [11,53-57]. Indeed, correlation between genetic distance and time was stronger for the VP60 dataset including the misdated AY269825 taxon than the RdRp dataset, which produced a more trustworthy substitution rate (r = 0.76 compared to r = 0.70). The decision to include or exclude taxa based on residuals from a best-fitting line is largely subjective, as there are no guidelines for how common large residuals are in tip-dated viral datasets. In fact, one of the AY269825-containing RHDV datasets had been subjected to this control prior to rate analysis, and the authors did not reject it as an outlier [12]. Finally, root-to-tip regression provides no insight into the magnitude of effect of any taxon on evolutionary estimates. The jackknife controls proposed here focus on detecting taxa that have had a disproportionate effect on the BEAST results, and, in the case of RHDV, offered strong quantitative evidence against including AY269825.

Another interesting finding is that without AY269825, the estimated substitution rate for the VP60 gene is almost identical to that of the RdRp gene, despite the fact that the latter dataset had fewer than half as many taxa (Table 1). Even while the RdRp dataset did not have as strong a temporal signal (Additional file 3), probably due to the lower number of taxa, it still produced a significantly more accurate substitution rate estimate than datasets with two to three times as many taxa that included just one misdated taxon (Table 1). Further, the estimated substitution rate from our VP60 RHDV + RCV dataset was nearly identical to that from another dataset which contained just 29 taxa (27 RHDV, 1 RCV, did not contain AY269825), including one distantly related European brown hare syndrome virus taxon as an outgroup [13]. This pattern of different sized datasets producing very similar substitution rates is not unique to RHDV. For example, two BEAST analyses of Dengue virus type 2 from datasets of 115 taxa and 67 taxa yielded nearly identical substitution rates [58,59]. Further, in the case of Human parechovirus, three BEAST analyses of three different genomic regions based on datasets with a range of 29–199 taxa also produced nearly identical substitution rates [55,60].

It is evident that assigning years of isolation to taxa should be done with great caution in tip-calibrated rate analyses. These results support favoring data sets with fewer taxa with verifiable dates of isolation over larger data sets with less quality control: additional good data do not swamp out the effects of one badly dated taxon. When researchers are including any ambiguously dated taxa, or when they want to be certain about the effects of each taxon on the rate analysis, jackknife controls provide a clear way to see these effects. As many sequences are added to GenBank without easily accessible papers describing in detail the isolation, passaging and sequencing of each isolate, it is necessary to verify if one or more of the sequences is having a disproportionate influence on the results. We propose n-1 jackknifing as one method for researchers using tip-calibrated analyses in BEAST to ensure that a small number of taxa are not spoiling their estimates.

## Methods

Full RHDV VP60 and RdRp gene sequences were downloaded from the GenBank Taxonomy Browser (http://www.ncbi.nlm.nih.gov/Taxonomy) on 11/16/11. Sequences for each gene with years of isolation available in GenBank or the literature were aligned manually in Se-Al v2.0a11 [61]. Those known to be genetically manipulated or extensively passaged in the lab prior to sequencing were removed from the datasets. Seven United Kingdom isolates that were identified as misdated modern contaminants in a previous ML analysis [12] were excluded because they only covered 30% of the full VP60 alignment.

As recombination events can lead to over-estimation of nucleotide substitution rates, each dataset was scanned for recombination using seven different algorithms (RDP, GENECONV, Bootscan, MaxChi, Chimaera, SiScan, and 3seq) implemented in RDP v3.44 [62]. Sequences with recombination signals detected by two or more algorithms were excluded from further analysis (EF558586 was excluded from both gene alignments as a potential recombinant).

GenBank accession numbers and dates of isolation for all taxa used in phylogenetic analyses are given in figures depicting the resulting trees.

### Complete dataset analyses

Modeltest v3.7 [63] was used to determine the best-fit model of nucleotide substitution for each of the alignments (by Akaike’s Information Criterion). Estimated nucleotide substitution rates and MCC trees for both the VP60 and RdRp datasets were obtained using BEAST v1.5.4 [64]. Each dataset was run for 200,000,000 generations using two different clock models (strict and uncorrelated lognormal) and three different demographic models (constant, exponential, and Bayesian skyline).The best-fitting clock/demographic model combination was determined using Bayes factors as implemented in Tracer v1.5 [65]. For each set of priors, two independent runs were performed to ensure that the results were replicable. For each dataset, a maximum likelihood analysis was performed using PAUP* v4.0b10 [66] to produce bootstrap-supported (1000 replicates) ML trees for comparison with the MCC trees. MCC trees for the complete VP60 and RdRp datasets, including alignments, are available in TREEBASE (http://purl.org/phylo/treebase/phylows/study/TB2:S12677).

RHDV has a divergent, avirulent sister group that may be conspecific, RCV. There were 39 (VP60) and two (RdRp) RCV sequences that were initially included in the complete gene datasets. Separate analyses were conducted using RHDV sequences alone.

Previous phylogenies of RHDV have identified four lineages, which are referenced here as lineages A-D, following the nomenclature of Kinnear and Linde (2010). Lineage A is referred to as group 3, lineage B is referred to as group 4, lineage C is referred to as group 2, and lineage D is referred to as group 1 in Kerr et al. (2009).

To test the strength of the temporal signal in the datasets, BEAST analyses were repeated an additional 10 times for each dataset with sampling years randomized [41,43,67], Additional file 6. The results of these tip-date randomization runs were then compared to the actual results to ensure that there was significant temporal structure present in the real datasets. Statistical significance was inferred from non-overlapping credibility intervals. In the instance of any overlap of 95% HPD intervals from the randomized datasets with 95% HPD interval from the actual dataset, a kind of post-hoc permutation test was employed. For all of the saved states (every 20,000 generations), excluding a 10% burn-in, the recorded mean substitution rate of the actual dataset was compared to those of each of the 10 randomized datasets. The probability that the two posterior distributions were the same was estimated by how often the mean rate from a randomized dataset exceeded the mean rate from the actual dataset. An additional test often used to assess rough temporal structure, root-to-tip regression analysis assuming a strict molecular clock, was conducted for the VP60 dataset using Path-O-Gen v1.3 [68].

### Control analyses

In the first attempt to identify if certain taxa were responsible for the substantial variation in published RHDV rate estimates based on the VP60 gene, the complete VP60 dataset (RHDV-only, 65 taxa) was used to generate 50 smaller datasets, each containing 30 taxa selected using a random number generator (Microsoft Excel, 2008). Each of these jackknifed datasets was run in BEAST, as described above, with the same best-fitting priors from the full dataset. In a parallel analysis, the 31 RdRp taxa were jackknifed into 30 independent subsets of 15 taxa each (exact taxa used for jackknife analyses available in Additional file 7). These smaller datasets were run until all parameters had stable ESS values (>200). Similarity between the mean estimated rates was determined visually by plotting the mean and 95% HPDs and by using the data to generate violin plots (violinmplot package in R [69,70]).

To further assess the effect of each individual taxon on substitution rate estimates, a jackknife “n-1” analysis was performed in which 65 datasets were generated from the complete VP60 dataset, each with one taxon removed. Each of these n-1 datasets was then run in BEAST, as described above with the same best-fitting priors from the full dataset. Whether a taxon had a statistically significant effect on substitution rate was determined by non-overlapping 95% HPD intervals with those from the complete VP60 dataset and from the other n-1 jackknife runs. An equivalent n-1 jackknife analysis was also performed on the RdRp dataset.

Any taxon identified as exerting a significant effect on a rate estimate was considered potentially misdated, and the method of leaf-dating via BEAST [71] was employed to determine a more accurate estimate of the suspect taxon’s age. Further, any such taxon was subsequently removed from the complete datasets, and the complete dataset analyses described above were repeated for the datasets without the suspect taxon.

## Competing interests

The authors declare that they have no competing interests.

## Authors’ contributions

ALH conducted all BEAST and PAUP* analyses and statistical analyses of the results. SD conducted the Path-O-Gen and post-hoc permutation analyses. ALH and SD wrote the paper. All authors read and approved the final manuscript.

## Supplementary Material

Additional file 1 **MCC tree produced from the complete RHDV + RCV VP60 dataset (without AY269825).** Node bars represent the 95% HPDs for the node ages. The dashed line indicates the 1984 emergence of RHD in China. Click here for file

Additional file 2 **MCC tree produced from the complete RHDV + RCV RdRp dataset.** Node bars represent the 95% HPDs for the node ages. The dashed line indicates the 1984 emergence of RHD in China. Click here for file

Additional file 3 **RHDV nucleotide substitution rates estimated from the tip-date randomization control analyses.** Mean substitution rates are shown with 95% HPD intervals for the VP60 dataset with AY269825 (65 taxa, left), the VP60 dataset without AY269825 (64 taxa, middle), and the RdRp dataset (31 taxa, right). For each group, the leftmost value is the estimated substitution rate from the actual dataset, while the following 10 values are those from the tip-date randomized datasets. Click here for file

Additional file 4 **RHDV nucleotide substitution rates estimated from the RdRp jackknife datasets.** Mean substitution rates are shown with 95% HPD intervals for each of the 30 datasets of 15 random taxa. Click here for file

Additional file 5 **RHDV nucleotide substitution rates estimated from the RdRp “n-1” jackknife datasets.** Mean substitution rates are shown with 95% HPD intervals for each of the 31 datasets. Each rate estimate is identified on the x-axis by the GenBank accession number and year of isolation of the taxon removed from its corresponding dataset. Click here for file

Additional file 6 **RHDV VP60 and RdRp Tip-Date Randomized Datasets.** Taxa are listed with the random dates assigned to them for each of the 10 tip-date randomized datasets for the full VP60, with and without AY269825, and the RdRp genes. Click here for file

Additional file 7 **RHDV taxa included in the VP60 and RdRp random jackknife control analyses.** Bolded taxa indicate those used in each of the 50 VP60 and 30 RdRp random jackknife datasets. Click here for file
