# Supplementary figures and images for: One misdated sequence of rabbit hemorrhagic disease virus prevents accurate estimation of its nucleotide substitution rate
Source: BMC Evol Biol. 2012 May 30;12:74. doi: 10.1186/1471-2148-12-74 (PMC3426481; doi:10.1186/1471-2148-12-74)

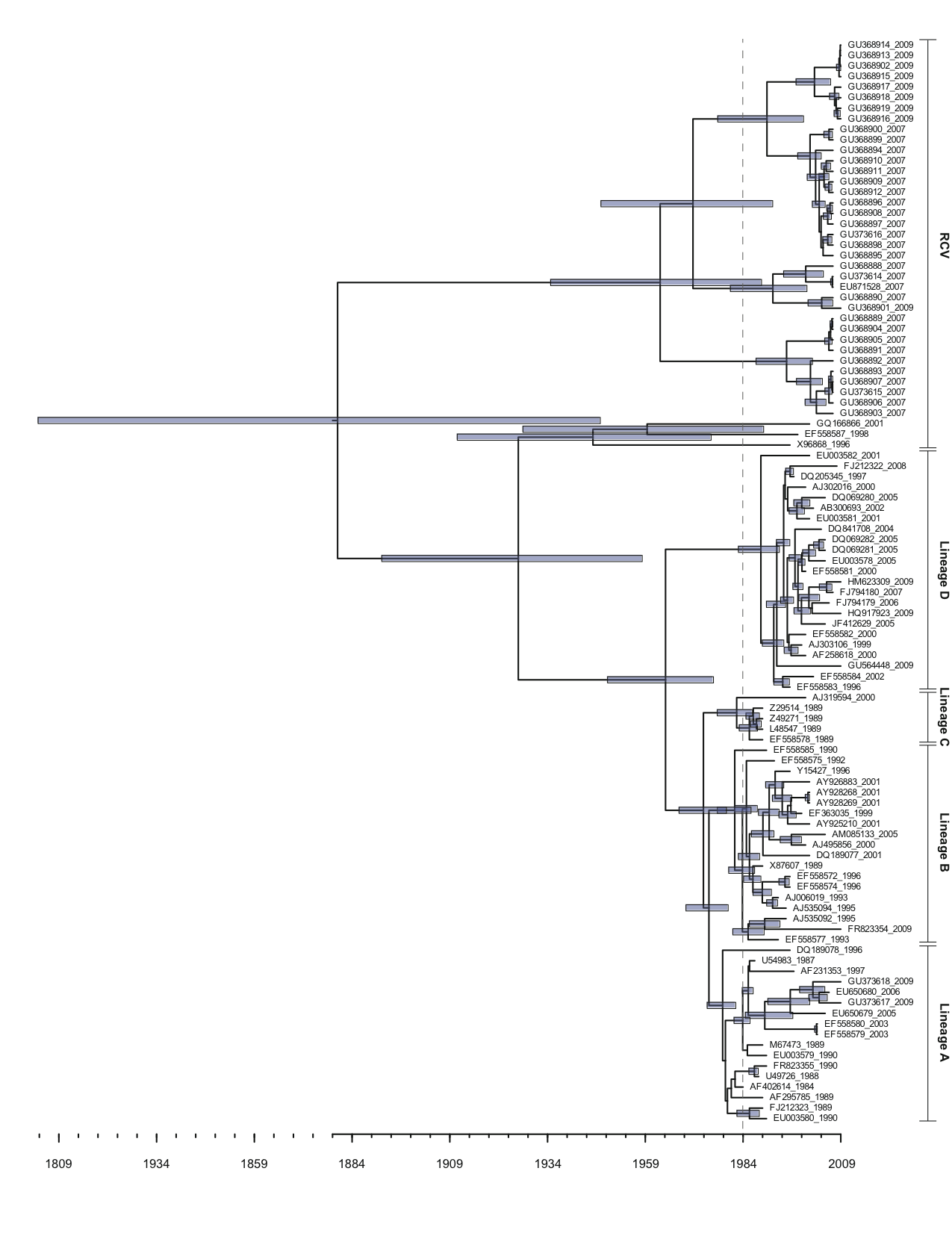

Supplement: Additional file 1 — MCC tree produced from the complete RHDV + RCV VP60 dataset (without AY269825). Node bars represent the 95% HPDs for the node ages. The dashed line indicates the 1984 emergence of RHD in China. [file 1471-2148-12-74-S1.tiff]

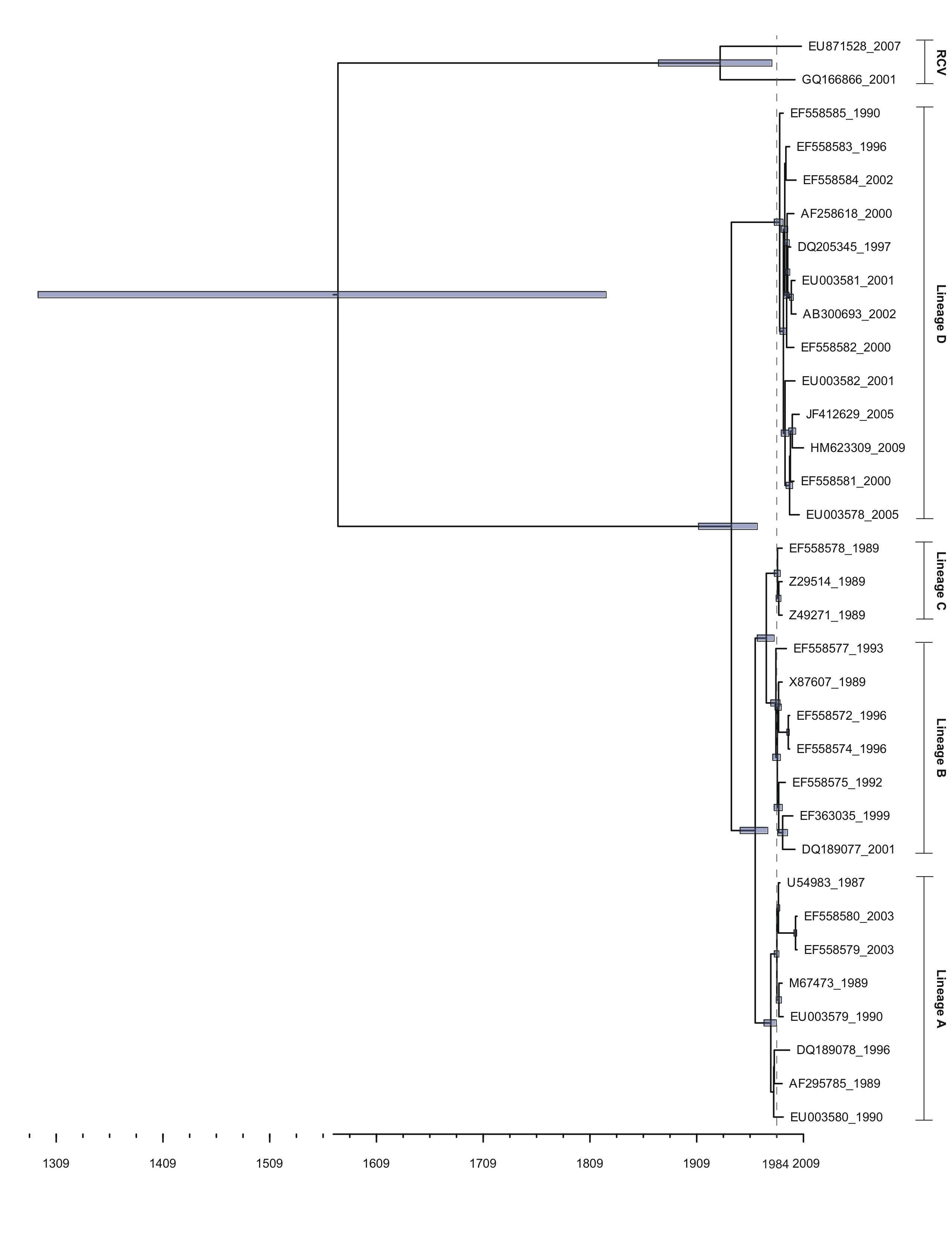

Supplement: Additional file 2 — MCC tree produced from the complete RHDV + RCV RdRp dataset. Node bars represent the 95% HPDs for the node ages. The dashed line indicates the 1984 emergence of RHD in China. [file 1471-2148-12-74-S2.tiff]

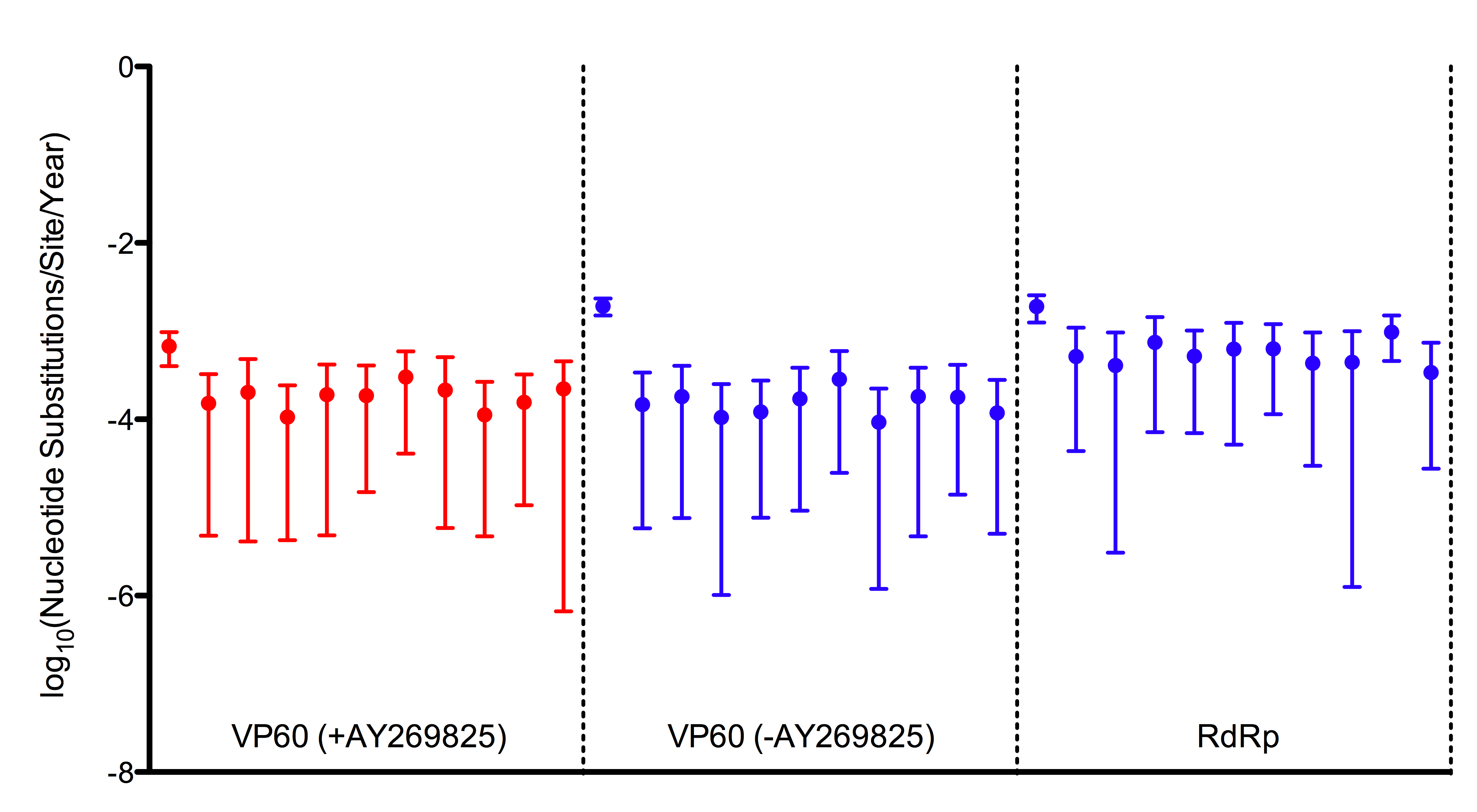

Supplement: Additional file 3 — RHDV nucleotide substitution rates estimated from the tip-date randomization control analyses. Mean substitution rates are shown with 95% HPD intervals for the VP60 dataset with AY269825 (65 taxa, left), the VP60 dataset without AY269825 (64 taxa, middle), and the RdRp dataset (31 taxa, right). For each group, the leftmost value is the estimated substitution rate from the actual dataset, while the following 10 values are those from the tip-date randomized datasets. [file 1471-2148-12-74-S3.tiff]

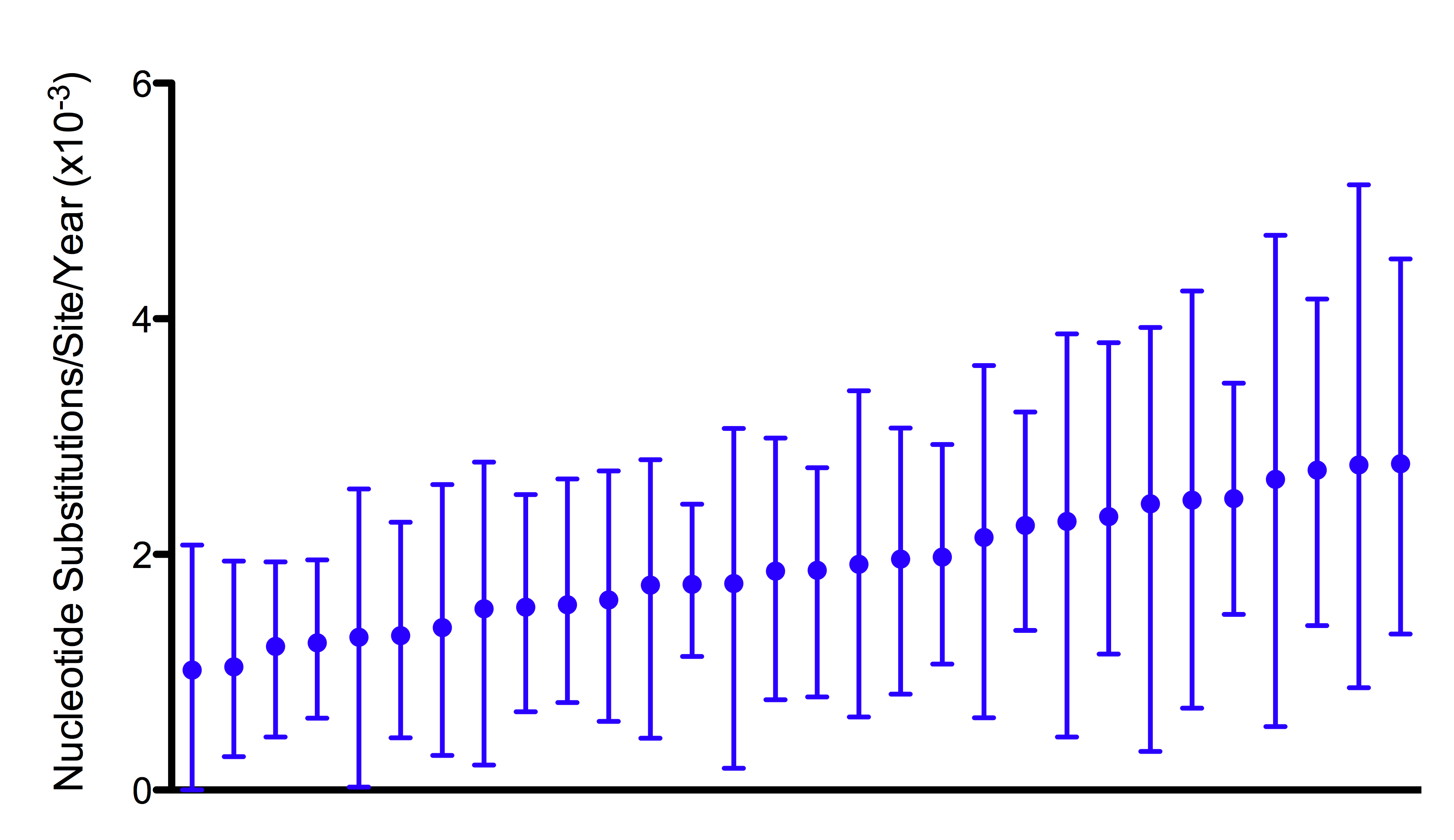

Supplement: Additional file 4 — RHDV nucleotide substitution rates estimated from the RdRp jackknife datasets. Mean substitution rates are shown with 95% HPD intervals for each of the 30 datasets of 15 random taxa. [file 1471-2148-12-74-S4.tiff]

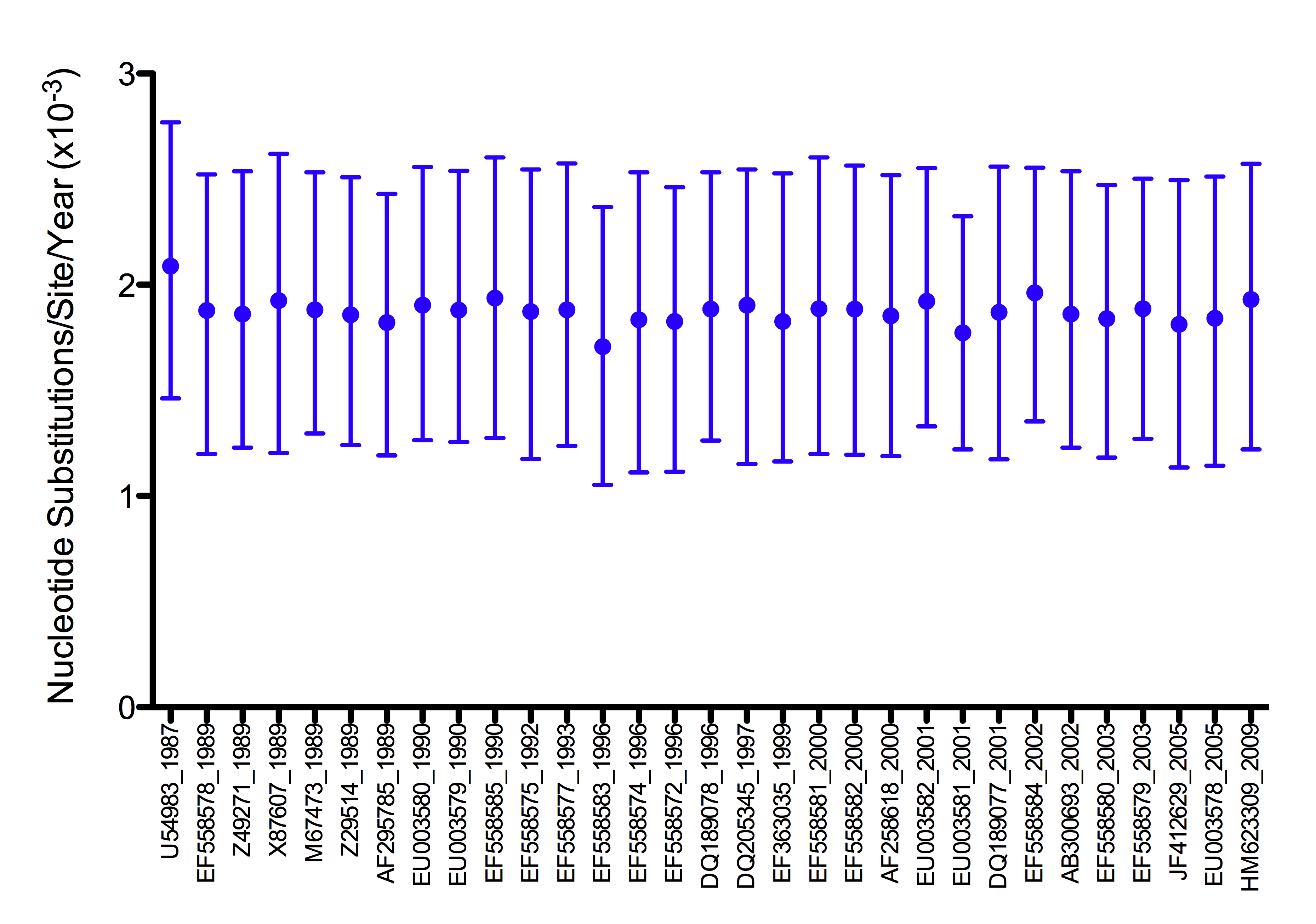

Supplement: Additional file 5 — RHDV nucleotide substitution rates estimated from the RdRp “n-1” jackknife datasets. Mean substitution rates are shown with 95% HPD intervals for each of the 31 datasets. Each rate estimate is identified on the x-axis by the GenBank accession number and year of isolation of the taxon removed from its corresponding dataset. [file 1471-2148-12-74-S5.tiff]
